# Mitigation strategies for heat-related illness during mass gatherings: Hajj experience

**DOI:** 10.3389/fpubh.2022.957576

**Published:** 2022-08-18

**Authors:** Yasir Almuzaini, Marriyah Alburayh, Ahmed Alahmari, Fahad Alamri, Abdulrahman Y. Sabbagh, Majid Alsalamah, Anas Khan

**Affiliations:** ^1^Global Centre for Mass Gatherings Medicine, Ministry of Health, Riyadh, Saudi Arabia; ^2^Emergency Medicine Administration, King Fahad Medical City, Riyadh, Saudi Arabia; ^3^Department of Emergency Medicine, College of Medicine, King Saud bin Abdulaziz University for Health Sciences, Riyadh, Saudi Arabia; ^4^Department of Emergency Medicine, College of Medicine, King Saud University, Riyadh, Saudi Arabia

**Keywords:** heat related illnesses, Haddon matrix tool, community risk reduction tool, combined model, mass gatherings, Hajj

## Abstract

**Introduction:**

To mitigate morbidity, mortality, and impacts of heat-related illnesses (HRIs) on health, it was vital to implement a comprehensive framework for HRI prevention and control. A recognized tool from the field of trauma prevention known as the Haddon matrix was applied. The matrix states that any event is affected by three factors: host, agent, and environment. In addition, another recognized tool known as the combined model was used in this study. The combined model is a three-dimensional model that includes the idea for the three axes of Haddon's matrix with the methodology of the community risk reduction (CRR) model.

**Aim of the study:**

To identify the environmental and individual risk factors of HRIs based on the Haddon matrix and the recommended prevention strategies by the CRR tool by using the combined model.

**Methodology:**

An extensive literature review was conducted to assess all the risk factors associated with HRI, as well as preventive measures. Then the Haddon matrix was used to structure, separating human factors from technical and environmental details and timing. After that, the combined model was used to set all responses and mitigation measures for each element obtained from the Haddon matrix tool.

**Conclusion:**

Projected increases in heat stress over the globe require the formulation and implementation of evidence-based HRI mitigation and preventive measures. In this study, we implemented the combined model that was utilized as a systematic strategy for the more theoretical framework of Haddon's matrix. Using the Haddon matrix to determine the HRI risk factors and the combined model to mitigate its impact was practical and helpful in planning, preparedness, and mitigating the HRIs during Hajj, provided a broad approach equivalent to the Swiss cheese model, and would facilitate an informed decision.

## Introduction

Heat-related illness (HRI) is when the body's core temperature exceeds its normal level, usually from exposure to an extremely high-temperature environment (1). HRI includes heat exhaustion (HE), heat injury, and life-threatening heat stroke (HS) (2). In addition, heat cramps and heat syncope are considered mild HRI and moderate HRI, respectively (3). Moreover, HE has mild signs and symptoms that might include fatigue, vomiting, and cramps. In contrast, heat injury and HS include organ damage and neurological alteration (1, 2). HRI has many risk factors, which can be either environmental or individual. The environmental factor includes high temperature, humidity, and heatwave, while the individual factors include age, hydration status, medications, pregnancy, obesity, and physical activity (acclimatization) (4, 5). HRI is responsible for at least 1,300 and 70,000 deaths per year in the United States and Europe (6). A systematic review conducted in 2012 found that a 1° increase in temperature could increase the number of individuals seeking medical care by 11% (6, 7). Hajj is a religious mass gathering event where Muslims worldwide gather in Makkah for pilgrimage (8). Since Makkah has a hot climate, especially during the summer seasons where the temperature could reach 40–50°C (9).

**Figure 1:**
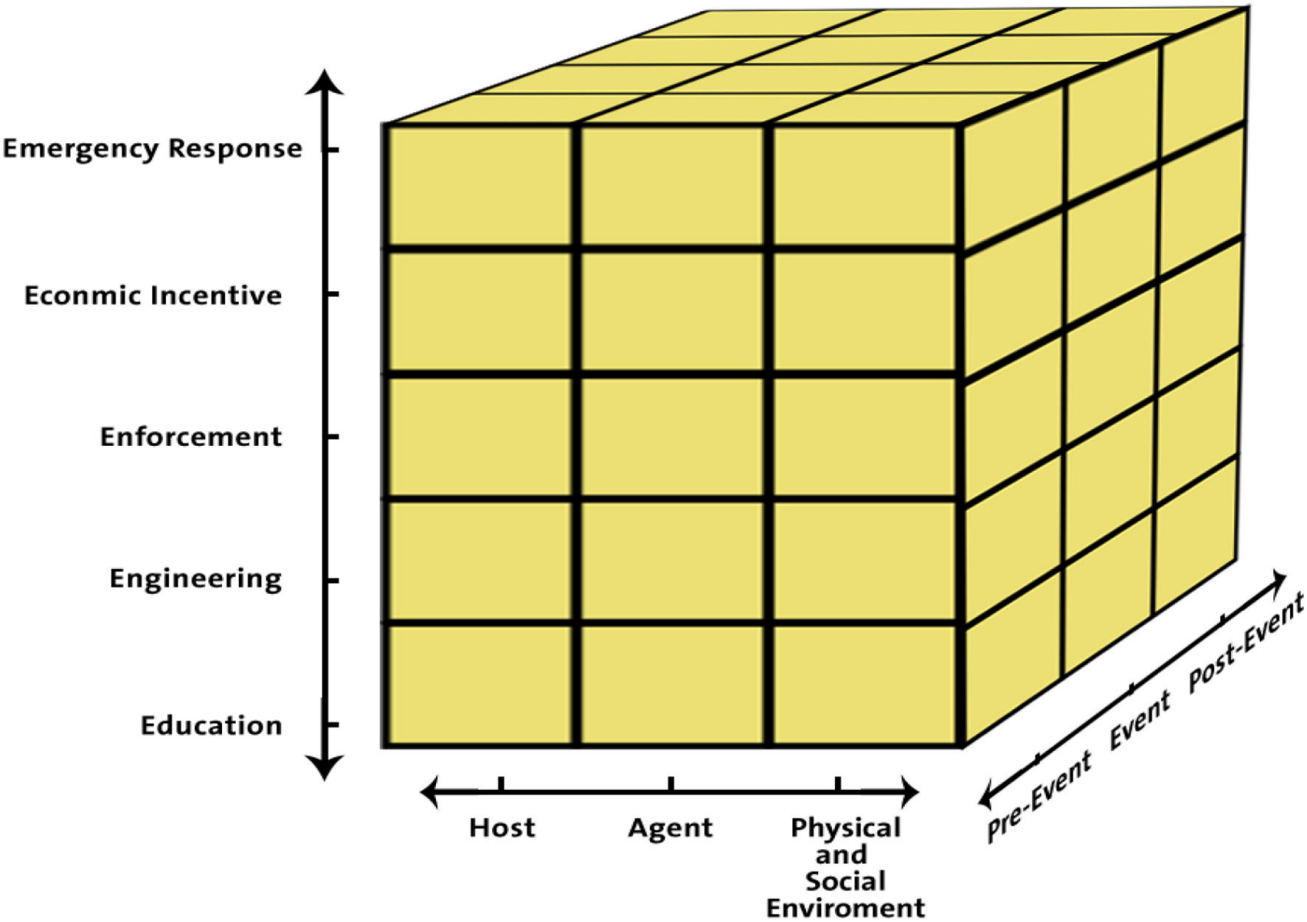
3D shape of the combined model.

A well-recognized tool known as the Haddon matrix in the field of injury prevention research and intervention was used in this study. Haddon matrix is a framework developed by Dr. William Haddon in 1968. The matrix might help to identify ways to modify these injuries. The Haddon matrix is constructed by arranging influencing factors: host, agent, and environmental factors (physical environment and socio-cultural environment). These factors are placed according to their influence before the event (pre-event), during the event itself, or after the event (post-event) (10). Even though, this tool was elaborated to use in trauma prevention, it has also been used for public health challenges (10). Another recognized tool known as the combined model was used in this study. The combined model is a three-dimensional framework containing the concepts for the three axes of Haddon's matrix using the Community Risk Mitigation Model methodology (community risk reduction; CRR) (11). This model includes (1) the three epidemiological factors, i.e., host, agent, and environments (social and physical), (2) the three-time periods of event occurrences classified as prior-event, during event, and post-event, (3) and systematic science-based methodology built on enforcement, education, economic incentives, engineering/environmental changes, and emergency response. Therefore, all the necessary elements for the understanding, comprehensive analysis, and management of HRIs are included in this three-dimensional model (12) (Figure 1). This study is aimed to identify the environmental and individual risk factors of HRIs based on the Haddon matrix and the recommended prevention strategies by the CRR tool by using the combined model.

## Methodology

An extensive literature review was conducted to assess all the risk factors associated with HRI, as well as preventive measures. Then the Haddon matrix was used to structure and separate human factors from technical and environmental details and timing. After that, the combined model was used to set all responses and mitigation actions for each element obtained from the Haddon matrix tool.

## Application of the Haddon matrix to HRIs vulnerability

### Pre-event: Factors affecting HRIs (Host)

Based on the findings in the Haddon matrix (Table 1), many factors could be considered as HRI morbidity and mortality determinates. For instance, negative changes in the thermoregulatory system have been associated with age, especially those over 60, making them more prone to heat effects (13). A cross-sectional study conducted during Hajj regarding HRI reported that 29% of the patients had suffered from heatstroke with mean age of 57.41 ± 12.35 years old and 67.75% had suffered from HE with mean age of 52.49 ± 17.70 years old (6). Furthermore, many studies showed that adverse impact of heat waves were more significant in women in all age groups in comparison to men (14). However, men have a higher risk of mortality due to HS, as more men work in a hot environment. Thus, physiological factors may contribute to these differences, but social factors appear to be the main ones (15). A pregnant woman is also more at risk of developing HRI when exposed to heat, mainly due to a rising core temperature and excessive metabolic heat production that occurs during pregnancy with less acclimatization reserve, where, heat exposure could lead to complications for both mother and child (16, 17). For instance, the mother could have a risk of uterine bleeding, hypertension, and eclampsia in the first three trimesters of her pregnancy (17). A systematic review conducted in 2020 on adverse birth outcomes presented that 9 out of 10 studies showed a significant correlation between heat exposure during pregnancy and adverse birth outcomes, (50%) involving preterm birth, (30%) on low birth weight, and (20%) on stillbirth (18). Notably, pre-existing comorbidities, such as diabetes, cardiovascular diseases, chronic lung disease, and central nervous system-related disorder, increase the risk of HRI and worsen the symptoms, leading to an increase in morbidity and mortality (6, 7, 13). In Hajj, most pilgrims are older individuals and many of them have pre-existing comorbidities, which are more likely to develop HRI complications (19). Drugs and medications interfering with body functions and mechanisms play a role in increasing HRI susceptibility, especially during excessive exercise and in extremely hot weather (7). For instance, drugs affect the nervous system as it controls sweating and thermoregulation (7, 17). Anticholinergic drugs are an example of these drugs as it affects sweating by blocking the action of a neurotransmitter called acetylcholine (ACh) (7, 17). These include drugs, such as hexamethonium and trimethaphan that inhibit ACh receptors and drugs, such as atropine or scopolamine, that inhibit muscarinic receptors, leading to blocking the sweat (anhidrosis) (20). However, a wide range of medications have anticholinergic effects with variation in the strength of their activities, yet most of the studies focused on a smaller group of them (17). Additionally, drugs that affect cardiac function, cutaneous blood flow or fluid homeostasis, adrenergic blockers, such as beta-blockers, antihypertensives, diuretics, laxatives, and antipsychotic drugs, and certain diet pills will also increase the risk of getting HRI (7, 17). Moreover, individuals with different body shapes and sizes respond to heat differently (21). Obesity is one of the risk factors leading to hyperthermia due to the increased ratio of body mass to the surface area, which minimizes body heat loss (22). In addition, obese individuals tend to have lower aerobic fitness hence their bodies direct most of the metabolic energy to produce heat instead of muscular action (17). Moreover, as the adipose tissue has half of the heat capacity of the lean tissue, it holds heat less effectively (17).

**Table 1:** Application of Haddon matrix on heat-related illnesses (HRIs).

**HRI**	**Agent**	**Host**	**Environment**	
			**Physical**	**Socio-cultural**
Pre	Heat Exposure	• Age • Gender • Co-morbidities • Medications • Lack of heat acclimatization • Population adaptations • BMI levels • Pregnancy • Race	• High temperature • Humidity • Transportation (walking to the place)	• Level of awareness • Education
During		• Dehydration • Exhaustion • Lack of using protective equipment (e.g., umbrella, sunblock)	• Availability of Shaded areas • Availability of Water sources • Congestion and Density of crowds • Transportation during the rituals	• Preferences of performing rituals during day • Perform rituals during the rush hours • (Religious restrictions) Male inability to wear head covers • Language barriers
Post		• HRIs cases (cramps, exhaustion, and stroke) • HRIs strategies and plans	• Availability and accessibility of medical care and transportation to medical care	• Medical care seeking

### Pre-event: Factors affecting HRIs (Physical environment)

#### High temperature and humidity

Heat-related illnesses are commonly a combination of external factors, such as exposure to hot weather, and internal factors, such as heat produced from physical exertion (17). Being exposed to a warm and humid environment might cause mild HRIs, such as heat cramps by tampering the fluid and salt imbalance, leading to contractions most commonly of the lower limbs muscles (1). Moreover, passive exposure to a warm and humid environment may also lead to moderate HRIs, such as HE or severe illnesses, such as HS (6). Additionally, it will endanger the individuals of having organ failures caused by the systematic inflammatory response to the heat exposure (7). Moreover, the mortality rate will increase once the temperature and duration of heat exposure increase (7).

#### Transportation (Walking distance)

Typically speaking, the human body is successful in maintaining its body temperature within a normal range of 35.8–37.3°C (23). For instance, pilgrims in Hajj are more likely to suffer from HRIs as they walk long distances under direct sunlight (1). In such a situation, the body temperature could increase 15–20 times more than at rest, so body core temperature could raise by 1°C every 5 min (23). The body core temperature in these settings (hot and humid environment) could reach 40°C causing central nervous system dysfunction and eventually a heatstroke (23).

### Pre-event: Factors affecting HRIs (Socio-cultural environment)

#### Knowledge, behavior, and level of awareness

Evidence showed that awareness of risk and behavioral adaptations are positively correlated (24). Such behaviors include refraining from direct sun exposure for hours, resting in cool-shaded places, and drinking more fluids (6, 25). Nevertheless, studies report that many pilgrims underestimate or are unaware of HRIs and are overzealous to perform religious rites during hot times of the day (26). About 19% of pilgrims are unaware of Makkah's weather and the potential consequences of high heat exposure before coming to Hajj (27). Moreover, 18–23% of pilgrims during the 2017 Hajj did not know that a high temperature can lead to illness or death (19). In addition, studies report that the vast majority of pilgrims are unwilling to change their Hajj plans, based on crowdedness or ambient temperature, and would follow their pre-planned Hajj schedules regardless of these factors (19).

### During the event: Factors affecting HRIs (Host)

#### Dehydration

Regular drinking of water is substantial to limit the severity of HRI. Therefore, the consumption of water intake regularly is a good way to maintain well hydration and avoid drinking water in case of thirst only (28). A study shows that consumption of 250 ml of fluid every 30–45 min leads to the prevention of HRIs. On the other hand, irregular water drinking and type of fluid intake have a high impact on the severity of the HRIs (28). However, early identification of the dehydration symptom may minimize the occurrence of these illnesses (6). Due to physiological changes because of aging, dehydration is more common in the older individual. These changes include debilitation of renal function, low total body water, and lower thirst sensation (29).

#### Lack of protective equipment

Heat-related illnesses can be minimized by using protective equipment, such as umbrellas, sunscreen, sunglasses, wearing light clothing, and using a fan (9, 30, 31). Alongside cooling transportation and accommodations are considered a part of protective measures (32). Lack of using these measures is a primer cause of heatstroke and HE among pilgrims (9). Studies have shown that pilgrims who do not practice HRI adaptation and prevention measures are at high risk of these illnesses (26). For instance, the use of umbrellas, hats, or sunscreen during Hajj is still substandard. Although not using umbrellas increased the risk of developing heat illnesses among pilgrims by more than 8-fold, studies have found that only 51–73% of Hajj pilgrims use umbrellas to protect themselves from the sun during Hajj (26). Likewise, less than 40% of pilgrims reported actually using sunscreen during the pilgrimage (26).

#### Levels of physical fitness

Lack of physical fitness is a crucial risk factor for HRIs, and it is expected to be low in Hajj as a significant percentage of pilgrims is old or obese (26, 33). A significant number of pilgrims are overweight or obese. A previous study conducted in Hajj in 1980 reported that most heatstroke and HE patients were overweight (34). Moreover, a large proportion of pilgrims are elderly, therefore, inferring a decline in muscular strength, endurance, flexibility, cardiorespiratory, and coordination and balance.

#### Overcrowding

While the weather generates most of the total heat load during Hajj, congested environments and heat re-emitted from mountains, asphalt and concrete surfaces, and crowds increase the heat load. The “penguin effect” is a concept related to the physiological changes that occur in the human body in crowded environments (35). In such environments, those in the center surrounded by heat-generating bodies tend to absorb the heat generated and cannot effectively dissipate the heat, leading to overheating and the possibility of HRIs. This effect can even occur when the ambient temperature does not seem high enough (35). Overcrowded Hajj accommodations can also increase heat stress, especially when the pilgrims stay on the plains of Mina. Such crowded settings increase the humidity and temperature inside the tents, thereby increasing heat stress for pilgrims. A previous study found that relative humidity levels in occupied tents ranged from 64–77% to 15–28% above outdoor levels, partly due to pilgrims' sweating. In addition, the study reported that pilgrims experienced very uncomfortable thermal conditions 38% of their time in the tent and difficult conditions the rest of the time (36).

## Mitigation strategies for HRIs using the combined model

The Saudi government has invested significantly in preventing HRIs and optimizing their management during the pilgrimage by improving Hajj infrastructure and services available to pilgrims. An overview of Saudi Arabia's response to the HRIs using the combined model is shown in Table 2. Education is the first step of the five key steps in the CRR model, and it is valuable in helping to improve individual cognition and adopt positive behaviors to reduce risk impact (11). Spreading risk awareness through various effective communication channels is a key factor in empowering vulnerable populations. For instance, many countries are introducing educational programs and pieces of training to raise awareness of HRI in their communities (37). Particularly, Saudi Arabia has tremendous efforts to enhance pilgrims' and health care workers' (HCWs) awareness regarding the risks of HRIs through implemented education campaigns pre and during the Hajj season (26). Alongside distributing brochures to the pilgrims upon arriving and establishing free phone lines are managed by trained HCWs (38). Moreover, the ministry of Hajj advises the agencies in different countries to enhance pilgrim's awareness before arriving at the holy sites by providing an awareness-raising program, thus to ensure that the pilgrims perform safe rituals (39). Environment engineering has also been adopted, this concept focuses on changing the environment to mitigate and control possible upcoming risks. For instance, the Saudi government has improved the Hajj services to prevent the HRI, such as building shaded areas alongside the availability of water mist sprays at the event (40). Furthermore, planting trees in open areas (41). Another significant investment is the availability of a metro system serving the pilgrims in the holy sites (42). The metro lines have been operated since 2010 for the 7 days of the rituals (42). In addition, air-conditioned buses are available during religious event (26). Moreover, temporary accommodation of more than 100,000 air-conditioned tents is available at the holy site (42). In addition, the tents used during the ritual events are fireproof (43). As Saudi Arabia's economy has changed, the health system in the country has gradually improved as well (9). Thus, the Saudi government has implemented numerous prevention strategies as economic incentives to reduce the impact of HRI during the Hajj season (19). For instance, umbrellas and free water are available and accessible to all pilgrims and in addition, water mist sprays are regularly available during religious event (19, 38). In addition, regular monitoring of the sacred water (Zamzam) is done to provide optimal source water quality (42). In 2021, the Saudi Data and Artificial Intelligence Authority (SDAIA) and the Doyof Al Rahman Program (DARP), in cooperation with Saudi Telecom Company (STC), launched a Pilgrim's Smart Bracelet (NUSK). The bracelets can demonstrate pilgrims' information and health status, measure the oxygen level and heartbeat, and display awareness messages (44). Effective public health emergency preparedness and response requires appropriate pre-event, during-event (crisis phase), and post-event (consequence phase) activities. Thus, in the context of emergency preparedness, the Saudi Ministry of Health (MoH) has provided various activities for each event phase. Furthermore, in cooperation with the Hajj committees, the Saudi MoH prepares for emergencies during Hajj by providing hospitals with the necessary equipment and trained personnel (6). The Saudi government provides free health services during the Hajj rituals through 16 hospitals and 128 primary health centers (PHCs), including seven seasonal health facilities and more than 13,000 core HCWs operate seasonal health facilities during the Hajj season (45). One of the essential health benefits provided is the cooling units, which provide rapid body cooling after exposure to extreme heat (9). Moreover, the Saudi MoH has developed HRI guidelines for HCWs (45). These guidelines were established in 2009 and updated in 2016 and 2019 (45). The guidelines consist of pre-hospital management and in-hospital management to properly recognize and handle HRI cases during the Hajj season (45). The pre-hospital management includes recognizing HRI cases, stabilizing the patient, and proper cooling. In contrast, in-hospital management includes confirming the diagnosis of HRI cases, in-hospital cooling, and supportive therapy (45). In addition, the Saudi MoH has also implemented health early warnings system surveillance (HEWS) (46). HEWS is using both event-based and syndromic surveillance (EBS) data to rapidly identify potential public health threats, trigger appropriate alerts for prompt epidemiological investigations, and monitor the trend of confirmed health issues, including HRI cases (46).

**Table 2:** Mitigation strategies using the combined model.

**Public health approach**	**Timeline**	**Mitigation strategies**		
		**Host**	**Physical environment**	**Socio-cultural**
**Education** Education influences audiences to refrain from risky or unhealthy behavior or take positive action to reduce risk.	Pre During Post	• Educational programs for the pilgrims/ healthcare workers	• Educational posters	• Increase pilgrims' awareness starting from their origin countries
**Enforcement** Enforcing legislation through inspections and fines for noncompliance.	Pre During Post	NA	• Regulations for responsible organization about housing conditions, sufficient water supply (Tawafa). • Crowds' times organization. • Ensure enough water supply during rituals. • Ensure safe housing conditions	NA
**Engineering/Environmental modification** Engineering includes incorporating new products and technology to modify the environment to prevent or control infection and deaths	Pre During Post	NA	• Increases shaded area • Ensure proper construction materials. • Green areas and water sprinkles • Traffic regulation	NA
**Economic incentives** Economic incentives are typically offered to encourage better choices and changes in behavior.	Pre During Post	• Offer free protective equipment's (e.g., Umbrella) • Offer mini free guides martials / pocketbooks	NA	NA
**Emergency response** Mitigate the effects of the infection and save lives.	Pre	• Surveillance system for HRIs cases • Preparation of emergency plans vehicles, staff, and equipment • Staff training		NA
	During Post	• Proper coordination between all involved entities • Following the proper guidelines for HRIs case management	• Availability of medical points • On-site medical care • Continuous evaluation of surge capacity plan • Stockpiles monitoring	

## Conclusion

Projected increases in heat stress over the globe require the formulation and implementation of evidence-based HRI mitigation and preventive measures. In this study, we implemented the combined model that was utilized as a systematic strategy for the more theoretical framework of Haddon's matrix. Using the Haddon matrix to determine the HRI risk factors and the combined model to mitigate its impact was practical and helpful in planning, preparedness, and mitigating of the HRIs during Hajj, provided a broad approach equivalent to the Swiss cheese model, and would facilitate an informed decision. The combined model provides a practical and comprehensive basis for the study and HRI mitigation strategies. The comprehensiveness of the combined model emphasizes coherence and evidence-based action. Therefore, the lessons of Hajj can also be applied to guide the policy-making and preventive actions of HRIs in the general population worldwide.

## Author contributions

YA contributed to conception and design of the study and manuscript writing. MAlb wrote the first draft of the manuscript. FA and AA: wrote sections of the manuscript. AK, MAlb, and AS contributed to manuscript revision, read, and approved the submitted version. All authors contributed to the article and approved the submitted version.

## Funding

This study received funding from Pfizer Saudi Arabia. The funder was not involved in the study design, collection, analysis, interpretation of data, the writing of this article, or the decision to submit it for publication.

## Conflict of interest

The authors declare that the research was conducted in the absence of any commercial or financial relationships that could be construed as a potential conflict of interest.

## Publisher's note

All claims expressed in this article are solely those of the authors and do not necessarily represent those of their affiliated organizations, or those of the publisher, the editors and the reviewers. Any product that may be evaluated in this article, or claim that may be made by its manufacturer, is not guaranteed or endorsed by the publisher.

## Abbreviations

HRIs: Heat related-illness
CRR: Community risk reduction.
